# Supplementary material for: A Scoping Review on the Economic Impacts of Healthy Ageing Promotion and Disease Prevention in OECD Member Countries
Source: Int J Environ Res Public Health. 2025 Jul 22;22(8):1161. doi: 10.3390/ijerph22081161 (PMC12385553; doi:10.3390/ijerph22081161)
Supplement: Supplementary file 1 [file ijerph-22-01161-s001.zip › ijerph-3696176-supplementary.pdf]

Supplementary Materials

Table S1. PRISMA extension for scoping reviews checklist.

Section	Item	PRISMA-ScR Checklist Item	Reported on Page #
Title			
Title	1	Identify the report as a scoping review.	1
Abstract			
Structured summary	2	Provide a structured summary that includes (as applicable): background, objectives, eligibility criteria, sources of evidence, charting methods, results, and conclusions that relate to the review questions and objectives.	1
Introduction			
Rationale	3	Describe the rationale for the review in the context of what is already known. Explain why the review questions/objectives lend themselves to a scoping review approach.	2
Objectives	4	Provide an explicit statement of the questions and objectives being addressed with reference to their key elements (e.g., population or participants, concepts, and context) or other relevant key elements used to conceptualize the review questions and/or objectives.	3
Methods			
Protocol and registration	5	Indicate whether a review protocol exists; state if and where it can be accessed (e.g., a Web address); and if available, provide registration information, including the registration number.	3
Eligibility criteria	6	Specify characteristics of the sources of evidence used as eligibility criteria (e.g., years considered, language, and publication status), and provide a rationale.	3
Information sources	7	Describe all information sources in the search (e.g., databases with dates of coverage and contact with authors to identify additional sources), as well as the date the most recent search was executed.	4
Search	8	Present the full electronic search strategy for at least 1 database, including any limits used, such that it could be repeated.	Table S3
Selection of sources of evidence†	9	State the process for selecting sources of evidence (i.e., screening and eligibility) included in the scoping review.	3
Data charting process	10	Describe the methods of charting data from the included sources of evidence (e.g., calibrated forms or forms that have been tested by the team before their use, and whether data charting was done independently or in duplicate) and any processes for obtaining and confirming data from investigators.	4
Data items	11	List and define all variables for which data were sought and any assumptions and simplifications made.	4
Critical appraisal of individual sources of evidence§	12	If done, provide a rationale for conducting a critical appraisal of included sources of evidence; describe the methods used and how this information was used in any data synthesis (if appropriate).	Not conducted
Synthesis of results	13	Describe the methods of handling and summarizing the data that were charted.	4

Results			
Selection of sources of evidence	14	Give numbers of sources of evidence screened, assessed for eligibility, and included in the review, with reasons for exclusions at each stage, ideally using a flow diagram.	18
Characteristics of sources of evidence	15	For each source of evidence, present characteristics for which data were charted and provide the citations.	5–9
Critical appraisal within sources of evidence	16	If done, present data on critical appraisal of included sources of evidence (see item 12).	Table S5
Results of individual sources of evidence	17	For each included source of evidence, present the relevant data that were charted that relate to the review questions and objectives.	15–16
Synthesis of results	18	Summarize and/or present the charting results as they relate to the review questions and objectives.	5–9
Discussion			
Summary of evidence	19	Summarize the main results (including an overview of concepts, themes, and types of evidence available), link to the review questions and objectives, and consider the relevance to key groups.	9
Limitations	20	Discuss the limitations of the scoping review process.	10
Conclusions	21	Provide a general interpretation of the results with respect to the review questions and objectives, as well as potential implications and/or next steps.	10–11
Funding			
Funding	22	Describe sources of funding for the included sources of evidence, as well as sources of funding for the scoping review. Describe the role of the funders of the scoping review.	11

Table S2. Eligibility criteria defined using the framework PICOST.

	Inclusion Criteria	Exclusion Criteria (Criteria Number)
Population	Individuals followed until their death in OECD member countries (the intervention is not limited to an age group)	Studies not reporting on individuals until their death (1)<br>Studies not conducted in OECD member countries (2)
Intervention	Disease prevention or health promotion interventions (including interventions on social determinants of health)	Studies not reporting on prevention or promotion interventions (3)
Comparison	No primary/secondary disease prevention or health promotion measures	No control group (4)
Outcome	Quantitative information on the net intervention economic effect, such as:<br>• narrow economic impacts: direct medical and non-medical costs, indirect costs<br>• broader economic impacts: public sector budget impact (changes to an individual's net transfers to the national budget over his/her lifetime, e.g., tax changes), macro-economic impacts (changes to national income or production)	Studies not reporting quantitative information on the net intervention cost (5)
Study design	Empirical studies<br>• interventional (RCTs, non-randomised controlled trials)<br>• observational (cohort studies, case–control studies)<br>Modelling studies<br>Reviews	Cross-sectional studies, case-series, case-reports, quasi-experimental studies (6)<br>Not a source of primary data (news, opinion article, commentary, editorial, methodology article) (7)<br>Duplicate articles (8)
Timeframe/miscellaneous	From 2016 to date<br>Papers in the English and French languages	Not retrieved (9)

Table S3. Summary of MEDLINE search strategy (via Ovid).

**Search Line**	**Search Terms: Keywords and Synonyms**	**Number of Hits**	**Search Line**	**Search Terms: Subject Headings**	**Number of Hits**
Promotion and prevention					
1	Preventi * OR promotion	2′230′174	2	primary prevention/OR secondary prevention/OR Preventive Medicine/OR exp Health Promotion/OR Risk Reduction Behavior/	149′508
Epidemiological impact					
3	(healthy ag#ing) OR (morbidity adj1 compression) OR (delayed morbidity) OR (pure ag#ing) OR (morbidity adj1 expansion)	2′460	4	aging/OR Healthy Aging/OR Morbidity/OR exp Health Status/	764′423
Economic impact					
5	Cost * OR expense * OR economic *	1′261′616	6	cost–benefit analysis/OR exp "cost of illness"/OR exp "Cost Control"/OR health care costs/OR health expenditures/OR capital expenditures/OR exp economics, dental/OR exp economics, hospital/OR exp economics, medical/OR economics, nursing/OR economics, pharmaceutical/OR external debt/OR exp taxes/	256′101
Combined results					
7	1 or 2				2′237′175
8	3 or 4				765′310
9	5 or 6				1′274′456
10 *	7 and 8 and 9 (limits applied)				1′796

* limited to records published in English or French, studies involving individuals in OECD member countries, and published from 2016 to date (search conducted on 8 May 2025).

Table S4. Data extraction table.

Study Identification	Comparison	Intervention Period	Methods	Discounting	Sensitivity Analyses Methods	Results Detailed	Sensitivity Analyses Results
Mihaylova, 2024[27]	No statin therapy	1980–2010	Participants were classified by preivous cardiovascular disease status, age, sex, LDL level, and 10-year cardiovascular disease risk. Microsimulation model developed using Cholesterol Treatment Trialits' Collaboration data the UK Biobank cohort. UK primary and hospital care data informed healthcare costs, and Health Survey for England data informed health-related quality of life. Meta-analyses of trials and cohort studies informed the effects of statin therapies on cardiovascular events, incident diabetes, myopathy and rhabdomyolysis.	3.5% annually for outcomes and costs	Deterministic (risk reduction in cardiovascular events with a statin, statin effect on incident cancer, compliance, treatment disutility, discount rate, effects of diseases on quality of life, costs) and probabilistic (treatment effects, quality of life, costs)	Standard statin therapy was cost-effective across all categories of people aged ≥40 years. Higher-intensity statin therapy was cost-effective among people 40–70 years old at higher cardiovascular disease risk (QRISK3 score ≥ 10%) or higher LDL level (≥4.1 mmol/L), and among people aged ≥ 70 years	Robust cost-effectiveness results except for the participants who were younger (age 40–49) and had low 10-year CVD risk (5–10%)
Hendy, 2024[21]	Family-based testing	Not reported	A decision tree–Markov hybrid model was used. Patients testing positive	3% annually for outcomes and costs	Deterministic (medication uptake, adherence and	ICER/QALY USD 202,718 and 571,939 for 20 and 35-year-olds, respectively	The ICER plane shows the intervention is cost-effective in 1.5% and 9.6% of

			for FH received a statin and after a year of adherence could begin PCSK9 inhibitor. WTP thresholds were sourced from patient surveys: USD 69,155/QALY.		persistence, genetic testing cost, genetic testing uptake, patient coinsurance) and probabilistic		simulations, for 20 and 35-year-olds, respectively
Altawalbeh, 2024[17]	No vaccination	Not reported	Costs were estimated using direct and indirect costs of acute illness and of pneumococcal-related long-term disability and mortality.	3% annually for effectiveness and costs	Deterministic (vaccination uptake, effectiveness, population health status, disease risk, case fatality, disability post disease, utilities, costs) and probabilistic	In the Black cohort, PCV20 was the dominant strategy; in the non-Black cohort, PCV20 cost USD 62,083/QALY, and PCV15/PPSV23 USD 1,2111,802/QALY	In probabilistic sensitivity analyses, PCV20 was favoured in 85.7% (Black) and 61.8% (non-Black) of model iterations at a USD 100,000/QALY gained WTP threshold
Kwon, 2023[28]	Usual care	Not reported	A dynamic fall–frailty feedback loop whereby falls influence long-term outcomes via frailty progression was used. Equity impacts were assessed through distributional cost-effectiveness analysis.	3.5% annually for outcomes and costs	Deterministic (discount rates, removing the falls-frailty feedback loop, frailty reduction, higher life expectancy, reduction in other-cause mortality risk gap across frailty categories) and probabilistic	ICER USD 26,269/QALY (95%UI USD 22,888 to 29,532). The intervention reduced inequality delineated by socioeconomic status quartile. Younger geriatric age groups could cross-subsidise their older peers for whom the intervention was cost-ineffective	The intervention had 93.4% probability of being cost-effective for the threshold of USD 28,736/QALY. Removing the falls–frailty feedback made the intervention no longer efficient or equitable

Liu, 2022[34]	No tax	2015–2115	A proportional multi-state life table-based Markov model was used. QALYs, the costs (intervention and healthcare costs), and the tax revenue were the main outcomes.	1.5% annually for outcomes and costs	Deterministic (CAD 0.01/oz and CAD 0.02/oz taxation) and probabilistic (mean BMI, RRs, the effect of change in energy intake on weight, free sugar intake and price elasticity of demand)	1.5 million QALYs gained and CAD 32,583 million saved in healthcare costs. ICER CAD −24933/QALY	The different tax levels remained cost-effective
Olchanski, 2021[14]	Usual care	Not reported	Microsimulation was used in the Diabetes Prevention Program trial population; 2 interventions were compared to usual care: lifestyle modification and metformin administration. For each intervention, targeted and non-targeted strategies were tested. The individualized risk of developing diabetes and projected diabetic outcomes were modelled. NMBs were estimated.	3% annually for outcomes and costs	Deterministic (QALY values, utility values of pre-diabetes and diabetes, metformin disutility, mortality rates, discounting)	Lifestyle modification conferred positive benefits and reduced costs for all risk quintiles. Metformin's NMB was negative for the lowest population risk quintile. Targeted administration of metformin (costs outweighing the benefits) conferred a benefit of USD 500/person. When prioritising individuals based on diabetes risk (20% of the target population), the difference in NMB ranged from USD 14,000 to 20,000/person	Metformin disutility had a strong effect in decreasing NMB of metformin, as well as the proportion of patients with positive NMB, increasing the value of targeting treatment
Visram, 2020[22]	No intervention	2015–2017	Behaviour change outcomes (client goals in relation to diet, physical	Applied as a sensitivity analysis	Deterministic (±10% concerning the extent of any	Total health gain of 288 QALYs. The overall net cost/QALY gained was	The result remained robust to discounting at 3.5% and

			activity, smoking) were recorded.		health gain and maintenance of behaviour change achieved)	USD 6770 and the total estimated societal value was at least USD 5.99 for every USD 1.74 spent on the service	changing all of the assumptions by ±10%
Soreskog, 2020[23]	Usual management	Not reported	Based on the SCOOP (screening for prevention of fractures in older women) RCT investigating whether community-based screening could reduce fractures, a Markov model was used	3.5% annually for outcomes and costs	Deterministic (discount rate, age, assuming that 100% of the excess mortality of fracture was related to the fracture event, assuming that screening had an effect only on the risk of hip fracture as the SCOOP study only analysed the hip fracture risk reduction) and probabilistic (treatment effect of screening)	Per patient, 0.015 QALYs (95%CI 0.007 to 0.023) gained and USD 452 (95% CI −915 to −122) saved	Deterministic: screening was cost-saving in all analyses. The cost/QALY was age-dependent. Screening became cost-neutral at the start age of 71 years. Probabilistic: the probability of screening being cost-effective at a WTP of USD 52,425 was 97%. In 87% of the simulations, screening was cost-saving
Liu, 2020[15]	Status quo	2018–2023	A validated microsimulation model (CVD-PREDICT) was used to estimate QALYs (policy effects on consumer diets and BMI-disease effects from published meta-	3% annually for outcomes and costs	Deterministic (energy intake compensation) and probabilistic (policy effect on consumer response and restaurant reformulation,	The consumer response alone was cost-saving, with net lifetime savings of USD 20.56 billion	Findings were robust across all analyses

			analyses), policy and health-related costs, and cost-effectiveness of the menu calorie labelling intervention, based on consumer response alone, and further accounting for potential industry reformulation. Change in calories were modelled to change in weight using an established dynamic weight-change model, assuming 50% of expected calorie reductions would translate to long-term reductions.		relation between changes in calories and weight, the association of changes in BMI with risk of CHF/stroke/diabetes, the individual CVD risk, costs, utilities		
Ananthapavan, 2020[31]	No intervention	2012–2018	Systematic literature reviews were undertaken to assess the evidence of intervention effectiveness on BMI and/or physical activity and their economic credentials. A multistate life table Markov cohort model was used.	3% annually for outcomes and costs	Deterministic (over 50 analyses) and probabilistic	All interventions were cost-effective. Eleven interventions were dominant. The ICER for the non-dominant interventions ranged from AUD 1728 to 28,703/HALY gained. Regulatory interventions tended to rank higher on their cost-effectiveness results, driven by lower implementation costs. The	The modelling results of each intervention were reported in the publications of individual studies; only the base case ICERs were presented in the paper

						programme-based policy interventions were generally based on higher quality evidence of intervention effectiveness	
Ratushnyak, 2019[18]	No screening	Not reported	Cost-effectiveness studies of breast, cervical, and colorectal cancer screening in the U.S. were identified using a systematic literature review. ICERs were updated by adding lifetime expenditures and health losses per QALY gained because of competing risks.	3% annually for outcomes and costs	Deterministic (cancer mortality was decreased instead of eliminating it. In the other sensitivity analysis, different QALY and cost estimates)	The impact of competing risks in life years gained incurred owing to screening were the highest for breast cancer and the lowest for cervical cancer. The updates suggest that ICERS are underestimated by USD 10,300 to 13,700/QALY gained. Cancer screening programmes that were considered cost-saving were found to be cost-effective	The results were robust to different assumptions with the exception of using cost values from a different study, which increased the costs/QALY by a factor of 2 (ICER remaining cost-effective)
Criss, 2019[19]	No screening	Not reported	By using shared inputs for smoking behaviour, costs, and quality of life, 4 independently developed microsimulation models evaluated the health and cost outcomes of annual lung cancer screening with LDCT. Different recommendations were tested: maximum age at	3% annually for outcomes and costs	Deterministic (cost variation for LDCTs and costs of lung cancer treatment, cost variation for persons younger than 65 years to address uncertainty regarding costs for these underrepresented	The 4 models showed that the NLST, CMS, and USPSTF screening strategies were cost-effective, with ICERs averaging USD 49,200, 68,600, and 96,700/QALY, respectively	NLST and CMS strategies had higher probabilities of being cost-effective (98% and 77%, respectively) than the USPSTF strategy (52%)

			which to stop lung cancer screening 80 years according to USPSTF, 77 according to CMS, and 74 according to NLST. ICERs with a WTP threshold of USD 100,000/QALY were measured.		persons in the Medicare data set, diminished screening adherence) and probabilistic (all cost and quality-of-life variables)		
Cleghorn, 2019[35]	Status quo	2011	A 250 mL serving size cap was modelled for all single serves (<600 mL) of SSBs in the National Nutrition Survey intake data. A multi-state life-table model used the change in energy intake and therefore BMI.	3% annually for outcomes and costs	Deterministic (discount rates, energy intake compensation, size caps)	The base case model (no compensation for reduced energy intake) resulted in an average reduction in SSB and energy intake of 23 mL and 44 kJ (11 kcal)/day or 0.22 kg of weight over a year. The total health gain and cost savings were 82,100 QALYs (95%UI 65,100 to 101,000) and NZD 1.65 billion (95%UI 1.19 to 2.24) over the lifespan of the cohort	Health gains and cost-savings associated with the less stringent 475 mL cap were lower (7030 QALYs and cost-savings of USD 136 m). Health gains associated with the 20% serving size reduction were 74,100 QALYs.<br>For 20% compensation of energy intake, QALY gains would be reduced to 71,700 QALYs; for 50%, 51,800 QALYs, and 100%, 18,400 QALYs
Bauer, 2019[29]	Standard care	Not reported	Decision–analytical Markov modelling was used. Individual models were developed for a selected range of health conditions, which had an expected high economic impact and for which	3.5% annually for outcomes and costs	Deterministic (probabilities for developing diseases, cohort entry ages, screening uptake and effectiveness,	Health checks led to a mean QALY gain of 0.074 (95%CI 0.072 to 0.119); and mean incremental costs of USD 8250 (95%CI 8225 to 8644). For a threshold of USD 51,690/QALY, health checks were not cost-	On the cost-effectiveness plane, at a threshold of USD 51,690/QALY, the vast majority of dots were centred at ICERs above USD 86,150.<br>The cost of an AHC was the only parameter that

			sufficient evidence was available for the modelling. Data from published studies as well as expert opinion informed parameters. Costs were expressed in 2016 GBP.		utilities, costs) and probabilistic	effective (mean ICER USD 147,585; 95%CI 142,637 to 227,149)	substantially influenced the results. If AHC could be provided at USD 86.15/person/year, then the probability of cost-effectiveness would be 70.1% (88.6%) at a cost per QALY threshold of USD 34,460 (51,690)
Stillwaggon, 2018[16]	No screening	Not reported	A decision–analytic model comparing costs of testing and treatment for mothers and infants was constructed with the lifetime societal costs without testing and the consequent morbidity and mortality due to lack of treatment or late treatment.	Discounting not reported for outcomes, costs discounted (discount rate mot reported)	Deterministic:<br>• for variables that have a range reported in the literature (prevalence among pregnant women, mother-to-child transmission risk, the cost of testing. risk of symptomatic Chagas, proportion of	Screening is cost-saving for all rates of congenital transmission greater than 0.001% and all levels of maternal prevalence above 0.06%. The value of lifetime societal savings due to screening and treatment is about USD 634 million for every birth year cohort	Universal screening is cost saving over a wide range of prevalence and screening costs in the United States (at any screening cost below USD 40/birth, the screening option is cost saving for any nonzero prevalence shown as low as 0.00032, which is 1/5 the actual estimated prevalence among all women giving birth annually in the US

					symptomatic Chagas that is cardiac, residual being digestive symptoms, likelihood of cure among adults after the acute phase), a range of values was used<br>• for other variables, ± 10% was used		
Pelczarska, 2018[38]	No screening	Not reported	Different strategies were analysed<br>• universal screening of people obtaining first job, using clinical diagnosis only or also genetic<br>• -universal screening of 6-	3.5% annually for outcomes; 5% annually for costs	Deterministic (FH prevalence, risk of CVD death in untreated FH patients, probability of performing cholesterol test, mean CVD treatment costs)	Screening ACS patients below 55/65 years of age is the most cost-effective strategy: the cost of one LYG amounts to EUR 100. Removing the age limit or using genetic tests reduced cost-effectiveness. All strategies remained cost effective: the cost of one QALY was EUR < 5040	FH screening was cost-effective in all scenarios

			year-olds, based on genetic diagnosis<br>• -opportunistic screening of people after the first onset of acute coronary syndrome or stroke, using clinical diagnosis only or also genetic; further split into: no age restrictions or limited to ACS before 55 in men and 65 in women.				
Pedersen, 2018[39]	No screening	Not reported	Screening strategies that varied by start age, frequency and test were analysed according to the vaccine received (bivalent/quadrivalent or nonavalent). Direct medical, transportation, and patient time costs were included. A commonly cited Norwegian WTP	4% annually for outcomes and costs	Deterministic (vaccine efficacy, cross-protection against non-vaccine-targeted HPV genotypes, compliance to screening and follow-up procedure, HPV test sensitivity, discount rate, direct medical costs only	The most cost-effective screening strategies were once per lifetime for nonavalent vaccinated women (EUR 20,720/QALY); twice per lifetime for bivalent/quadrivalent vaccinated women (EUR 53,570/QALY). The cost gains compared to the current screening guidelines were EUR	Bivalent/quadrivalent vaccination were the most sensitive to cross-protection against non-vaccine-targeted HPV genotypes, screening compliance, inclusion of medical costs only, and no discounting. For nonavalent vaccination, once-only screening remained the most cost-effective strategy except for the reduction in vaccine

			threshold of EUR 75,000/QALY was used.		and productivity losses associated with sick leave)	599/bivalent/quadrivalent vaccinated woman, and EUR 725/nonavalent vaccinated woman. For the first cohort of 22,000 fully vaccinated women in 2009, EUR 13.2 million for the bivalent/quadrivalent and EUR 16.0 million for the nonavalent vaccinated would be saved	efficacy and no discounting, for which twice per lifetime screening was the most cost-effective strategy
Jonsson, 2018[36]	Current clinical practice	Not reported	The model simulates the individual patients considered for treatment. Information on current management of osteoporosis in terms of patient characteristics and treatment patterns were derived from a Swedish osteoporosis research database based on national registers and patient records.	3% annually for outcomes and costs	Deterministic (treatment persistence increased, patients who discontinue treatment switch to next line of treatment, guidelines fully implemented)	QALY gains 14,993, cost savings EUR 776 M	Cost/QALY ranged from cost-saving to EUR 31,368 depending on the scenario.
Cleghorn, 2018[37]	No tax increases	2011–2020	A multistate life table model with 16 parallel tobacco-related diseases was used, parameterised with rich national data by sex, age and ethnicity. The net health system cost was the cost of a	3% annually for outcomes and costs	Probabilistic	QALY gains 39,100 (95%CI 20,900 to 67,300), cost savings NZD 799 million (95% CI 433 to 1350 million). The health gains associated with a tobacco tax come mainly from reducing the incidence of	See data in adjacent left column.

			new law to legalise a tobacco tax and any difference in projected future health system expenditure.			COPD, lung cancer, heart disease and stroke. These diseases are more prevalent among the Māori population (which is younger and has high smoking prevalence), hence the greater per capita benefit for Māori aged 20–65 years	
Wilson, 2017[40]	Standard care	Not reported	Intervention effectiveness estimates came from a systematic review. New Zealand specific intervention costs were extracted from a RCT.	3% annually for outcomes and costs	Deterministic (discount rate) and probabilistic	ICER USD 4652/QALY (95% UI cost-saving to USD 13,003/QALY). Targeting the intervention to people aged 75 years and older with previous injurious falls was cost-saving (median ICER USD −74/QALY, 95%UI cost-saving to USD 3909/QALY)	The ICER remained cost-saving to cost-effective
Neumann, 2017[41]	No intervention	Not reported (duration 5 years)	Indirect costs were included (sickness absence, early retirement, production loss due to mortality). The Saxon Diabetes Prevention Program in Germany, was used to determine intervention costs (identifying individuals at higher risk for	3% annually for outcomes and costs	Deterministic (different scenarios by sex and age groups, possibility of changing from T2D to pre-diabetic states removed, states' and intervention cost varied by ±10%, the time the	ICERs ranged from USD 5313/QALY gained (women, 30 years) to USD 12,775/QALY gained (men, 70 years).	The CEA curves showed that the probability of the intervention being cost-effective at the threshold value of USD 88,303/QALY gained was very high for all scenarios, ranging from 85.0 to 91.1%.

	developing T2D, weekly courses for 8 weeks on the physiology of the body, healthy eating, exercise and motivation guided by prevention managers, follow-up mentoring with the prevention manager as long as the participant wishes; prevention managers were individuals trained for motivational counselling and diabetes prevention). The success of the intervention was defined by changes in 3 characteristics: weight reduction of 5%, increase in physical activity by 13%, and an increase by 10% of the proportion of individuals consuming at least 5 portions of fruits and vegetables per day (changes described as realistic due to lifestyle intervention programmes). It was assumed the effectiveness of the	intervention showed effect doubled, the weight reduction was changed to 3.3% which corresponds to the average decreased weight in community-based programmes in the US, the weight reduction of 3.3% was assumed to be the only effect) and probabilistic (transition probabilities, costs)	

			intervention decreased over time.				
Crino, 2017[33]	Status quo	2010 to the individuals' deaths	Health outcomes were modelled from calculated changes in BMI.	3% annually for outcomes and costs	Deterministic (government-imposed legislation versus voluntary industry pledge, no compensatory eating versus compensatory eating, percentage of individuals concerned by compensatory eating) and probabilistic	HALYs gained 218,454 (95%UI 166,088 to 286,112), cost savings AUD 2.2 billion (95%UI 1.2 to 2.5 billion)	Dominant intervention in all scenarios
Cobiac, 2017[30]	Status quo	2010 to the individuals' deaths or age 100	Taxes on saturated fat, salt, sugar, and SSBs, and a subsidy on fruits and vegetables were simulated. Dietary-related diseases, their mortality, and mortality from all other causes were modelled using food prices elasticities. The sizes of the taxes and subsidy were set such that when combined as a package, there would be a negligible effect on average weekly	3% annually for outcomes and costs	Deterministic (constraints on changes in total energy intake and total weight of foods consumed, food industry reformulation of foods to avoid taxes, under- or over-shifting of price changes on taxed products) and probabilistic (food consumption, proportion of	The combination of taxes and subsidies might avert as many as 470,000 DALYs (95% UI: 420,000 to 510,000) in the Australian population of 22 million, with a net cost-saving of USD 3.16 billion (95% UI: USD 2.2 billion to USD 4.3 billion). The sugar tax produced the biggest estimates of health gain (270,000 [95% UI: 250,000 to 290,000] DALYs averted), followed by the salt tax (130,000 [95% UI:	The combination of all 5 tax and subsidy interventions had a 100% probability of cost-savings under all scenarios. The subsidy intervention alone was dominated, with only an 11% probability of being cost-effective against a USD 46,556/DALY threshold.

			expenditure on food (<1% change). Costs were presented in 2010 AUD.		purchased food that is wasted, food price elasticities, relative risks of disease)	120,000 to 140,000] DALYs), the saturated fat tax (97,000 [95% UI: 77,000 to 120,000] DALYs), and the sugar-sweetened beverage tax (12,000 [95% UI: 2100 to 21,000] DALYs). The fruit and vegetable subsidy (−13,000 [95% UI: −44,000 to 18,000] DALYs) was a cost-effective addition to the package of taxes. The subsidy did not necessarily lead to a net health benefit for the population when modelled as an intervention on its own because of the possible adverse cross-price elasticity effects on consumption of other foods (e.g., foods high in saturated fat and salt)	
Breeze, March 2017[25]	Status quo	Not reported	Evaluation of the following interventions: soft drinks taxation (20% tax if SSB), retail policy in socially deprived areas (opening of a supermarket), workplace intervention (healthy	1.5% annually for outcomes and costs	Deterministic (intervention effects and duration, disease incidences, diagnostic thresholds, effects of diseases on other diseases such as	All interventions generate better health outcomes and cost savings. Net lifetime benefit per individual: soft drinks tax USD 18.71, retail policy USD 18.50, workplace intervention USD 4.65, community	High-risk population screening and intensive lifestyle intervention had the highest probability (78%) of being the most cost-effective strategy

			eating promotion in the canteen), community-based intervention (men-only cooking skills and weight-loss programme), screening of high-diabetes-risk individuals and intensive lifestyle intervention. Employment costs were also evaluated.		diabetes on stroke, costs, utility values, depression and work absence associated with the onset of diabetes versus the diagnostic, rate of weight regain, uptake rates of the interventions, discount rate) and probabilistic	intervention USD 2.57, high-risk population intervention USD 64.30	
Breeze, January 2017[24]	No intervention	Not reported (duration 1 year)	An individual patient simulation model was used in 6 different high-risk subgroups for diabetes (age 40–65 years, low SES, HbA1c > 6%, Finnish Diabetes Risk score > 0.1, BMI ≥ 35kg/m^2, South Asian). The model incorporates trajectories for HbA1c, 2 h glucose, fasting plasma glucose, BMI, systolic blood pressure, total cholesterol and HDL cholesterol. Patients can be diagnosed with diabetes, cardiovascular disease, microvascular	1.5% annually for outcomes and costs	Deterministic (intervention and states' costs, utilities, statin uptake, no BMI effect on cancer or osteoarthritis or depression, no diabetes or stroke effect on depression, CVD incidence, diabetes threshold, CHF incidence, discount rate) and probabilistic	The intervention produces 0.0003 to 0.0009 incremental QALYs and saves up to USD 1.68/person in the general population, depending on the targeted subgroup. Cost-effectiveness increases with intervention intensity. The most cost-effective options are to target individuals with HbA1c > 6% or with a high Finnish Diabetes Risk probability score	Deterministic: the intervention remains cost-effective in all population subgroups; in all cases the HbA1c > 6% subgroup remains the most cost-effective<br>Probabilistic: the intervention is highly likely to be cost-effective in all subgroups at a threshold of USD 32,174/QALY. The cost-effectiveness acceptability curve indicates that the HbA1c > 6% group has a high probability of cost-effectiveness

			complications of diabetes, cancer, osteoarthritis and depression. Other causes of mortality are modelled, but the costs of competing risks are not. Low-, medium-, and high-intensity interventions were modelled. Outcomes were averaged across the whole population simulated rather than just the intervention groups.				
Zur, 2016[42]	No screening	Not reported	AUDIT was used as the screening tool; 18 alcohol-related causes of death were modelled.	3% annually for outcomes and costs	Deterministic (intervention frequency, uptake percentage, effectiveness, costs, discount rate)	ICER USD 8729/QALY	The intervention remained cost-effective despite a lower intervention uptake. Changing the intervention cost (from USD 16.54 to 42.01) and effectiveness (reduction in consumption from −23.5 to 6.7% for men, −22.1 to −13.3% for women) resulted in a cost-ineffective intervention (ICER USD 35,575/QALY)
Veerman, 2016[32]	Status quo	2010 to the individuals' deaths	A 20% tax on SSBs was applied; participants were adults (≥20 years old) in 2010. It was	Applied as a sensitivity analysis	Deterministic (expected BMI trend, duration of the effect on BMI,	Over the lifetime of adult Australians alive in 2010, seemingly modest estimated changes in BMI	In all scenarios, the policy was likely to be cost-saving from a health sector perspective.

			assumed that producers would pass on the price increase in full to the consumers. Obesity-related disease, mortality, and healthcare expenditure were modelled. Healthcare costs for non-obesity related diseases were included, so unrelated healthcare costs in added years of life are accounted for.		pass-on rate, discounting of outcomes and costs) and probabilistic (intervention effect on mean BMI, relative risks of incident disease, intervention costs)	as a result of the SSB tax translated to gains of 112,000 HALYs for men (95% UI: 73,000-155,000) and 56,000 (95% UI: 36,000–76,000) for women, and a reduction in overall healthcare expenditure of USD 567 million (95% UI: 343 million- 810 million). The tax is estimated to reduce the number of new type 2 diabetes cases by approximately 800/year. Twenty-five years after the introduction of the tax, there would be 4400 fewer prevalent cases of heart disease and 1100 fewer persons living with the consequences of stroke, and an estimated 1606 extra people would be alive as a result of the tax. The tax is estimated to generate USD 371 million in revenue each year.	
Razdan, 2016[20]	No intervention	2010–2012	The study was conducted from the perspective of a regional Organ Procurement Organization (OPO).	3% annually for outcomes; no cost discounting	Deterministic (probability of a registrant becoming a donor, an individual	6708 individuals joined the organ donor registry (95%CI 5429 to 7956) at a cost of USD 455/registrant (95%CI 383 to 562). The	Not reported

			Promotion costs were obtained from the OPO. The number of new registrants was obtained from the OPO and the departments of motor vehicles that maintain the donor registry. The value of registrants in terms of organ donors was computed based on a registrant's age-dependent mortality and probability of becoming an organ donor.		becoming a donor without joining the registry, leaving the registry, dying, dying by an eligible death; promotion effectiveness; age of joining the registry; discount rate)	registrants resulted in 8.2 future donors equivalent to 4.2 present-day donors (95%CI 2.5 to 6.6) at a cost of USD 72,6000 (95%CI 462,000 to 1.2 million). The cost per donor is less than society's WTP of USD 1,086,000 based on the value placed on 1 QALY (USD 100,000)	
Lomas, 2016[26]	No intervention	2013–2016	The health impact was calculated as a one-off benefit (childhood asthma prevalence reduction) and annual benefit (effect on all-cause death, coronary events, low birthweight births, preterm births). NHS and Personal Social Services costs were used.	3.5% annually for outcomes and costs	Not conducted	Cost of implementation USD 11.07 million, generating a one-off benefit of USD 5.80 million and an annual benefit of USD 3.69 million	Not conducted

Table S5. Quality appraisal of included economic evaluations using the Drummond 10-item checklist.

Study	Research Question Well-Defined?	Comprehensive Description of Alternatives?	Effectiveness of Programme Established?	Important and Relevant Consequences for Each Alternative Identified?	Costs and Consequences Measured Appropriately?	Costs and Consequences Valued Credibly?	Costs and Consequences Adjusted for Differential Timing?	Incremental Analysis of Costs and Consequences Performed?	Allowance Made for Uncertainty in Estimates?	Presentation and Discussion of Study Results include all Issues of Concern to Users?
Mihaylova, 2024[27]	✓	✓	✓	✓	✓	✓	✓	✓	✓	✓
Hendy, 2024[21]	✓	✓	✓	✓	✓	✓	✓	✓	✓	✓
Altawalbeh, 2024[17]	✓	✓	✓	✓	✓	✓	✓	✓	✓	✓
Kwon, 2023[28]	✓	✓	✓	✓	✓	✓	✓	✓	✓	✓
Liu, 2022[34]	✓	✓	✓	✓	✓	✓	✓	✓	✓	✓
Olchanski, 2021[14]	✓	✓	✓	✓	✓	✓	✓	✓	✓	✓
Visram, 2020[22]	✓	✓	✓	✓	✓	✓	✓	✓	✓	✓
Soreskog, 2020[23]	✓	✓	✓	✓	✓	✓	✓	✓	✓	✓
Liu, 2020[15]	✓	✓	✓	✓	✓	✓	✓	✓	✓	✓
Ananthapavan, 2020[31]	✓	✓	✓	✓	✓	✓	✓	✓	✓	✓
Ratushnyak, 2019[18]	✓	✓	✓	✓	✓	✓	✓	✓	✓	✓
Criss, 2019[19]	✓	✓	✓	✓	✓	✓	✓	✓	✓	✓
Cleghorn, 2019[35]	✓	✓	✓	✓	✓	✓	✓	✓	✓	✓
Bauer, 2019[29]	✓	✓	✓	✓	✓	✓	✓	✓	✓	✓
Stillwaggon, 2018[16]	✓	✓	✓	✓	✓	✓	X	✓	✓	✓
Pelczarska, 2018[38]	✓	✓	✓	✓	✓	✓	✓	✓	✓	✓
Pedersen, 2018[39]	✓	✓	✓	✓	✓	✓	✓	✓	✓	✓
Jonsson, 2018[36]	✓	✓	✓	✓	✓	✓	✓	✓	✓	✓
Cleghorn, 2018[37]	✓	✓	✓	✓	✓	✓	✓	✓	✓	✓
Wilson, 2017[40]	✓	✓	✓	✓	✓	✓	✓	✓	✓	✓
Neumann, 2017[41]	✓	✓	✓	✓	✓	✓	✓	✓	✓	✓
Crino, 2017[33]	✓	✓	✓	✓	✓	✓	✓	✓	✓	✓
Cobiac, 2017[30]	✓	✓	✓	✓	✓	✓	✓	✓	✓	✓
Breeze, March 2017[25]	✓	✓	✓	✓	✓	✓	✓	✓	✓	✓
Breeze, January 2017[24]	✓	✓	✓	✓	✓	✓	✓	✓	✓	✓

Zur, 2016[42]	✓	✓	✓	✓	✓	✓	✓	✓	✓	✓
Veerman, 2016[32]	✓	✓	✓	✓	✓	✓	✓	✓	✓	✓
Razdan, 2016[20]	✓	✓	✓	✓	✓	✓	✓	✓	✓	✓
Lomas, 2016[26]	✓	✓	✓	✓	✓	✓	✓	✓	X	✓
